# Knowledge, attitudes, and practices toward African swine fever among commercial pig farm producers in Takeo and Kampong Speu provinces, Cambodia

**DOI:** 10.3389/fvets.2026.1889657

**Published:** 2026-08-14

**Authors:** Bunna Chea, Kouch Theng, Ha Thi Thu Le, Bao Dinh Truong, Flavie Luce Goutard

**Affiliations:** 1 Faculty of Veterinary Medicine, Royal University of Agriculture, Phnom Penh, Cambodia; 2 CIRAD, UMR ASTRE, Hanoi, Vietnam; 3 Vietnamese Institute of Animal and Veterinary Science, Hanoi, Vietnam; 4 Faculty of Animal Science and Veterinary Medicine, Nong Lam University, Ho Chi Minh, Vietnam; 5 ASTRE, CIRAD, INRAE, Montpellier University, Montpellier, France

**Keywords:** African swine fever, attitudes, commercial pig farms, knowledge, practices

## Abstract

African swine fever (ASF) poses a major transboundary threat to swine production in Cambodia and across the Asia–Pacific region, with significant economic and food security implications. A cross-sectional survey was conducted from August to October 2024 to assess the knowledge, attitudes, and practices (KAP) of commercial pig producers in Kampong Speu and Takeo provinces regarding ASF prevention and control. A structured questionnaire was administered to 76 commercial farms housing more than 100 fattening pigs or 50 breeding pigs. Descriptive statistics were used to summarize KAP levels, biosecurity implementation, and challenges, while inferential analyses explored associations among KAP components. Scores were categorized according to Bloom's cut-off criteria as poor (< 60%), moderate (60%−80%), or good (>80%). Respondents generally demonstrated moderate knowledge of ASF characteristics, transmission pathways, and prevention and control measures (73.6%; 95% CI: 64.2–75.5). Nonetheless, notable gaps remained, particularly in recognizing clinical signs and understanding diagnostic procedures ([41.4%; 95% CI: 34.6–49.7). Most producers expressed positive attitudes toward ASF-related biosecurity measures (89.4%; 95% CI: 80.6–94.6) and reported high compliance with hygiene protocols and equipment disinfection (88.3%; 95% CI: 79.0–93.6). No significant associations were found between knowledge and either attitude or practice (*p* > 0.05), and sociodemographic factors did not significantly influence KAP outcomes (*p* > 0.05). In conclusion, commercial pig producers demonstrated moderate knowledge and generally positive attitudes toward ASF prevention, with strong implementation of several routine biosecurity and hygiene practices. However, important gaps remained in ASF reporting, visitor-log recording, and emergency preparedness knowledge. To achieve sustainable control, interventions should move beyond information dissemination and promote behavioral change through targeted education, continuous veterinary engagement, and practical biosecurity support. Strengthening these components will be essential for enhancing ASF preparedness and resilience within Cambodia's commercial pig sector, thereby mitigating the risk of disease spread.

## 1 Introduction

African swine fever (ASF) is a highly contagious viral disease that affects both domestic and wild pigs. Although experimental or limited field use of ASF vaccines has begun in some countries (e.g., Vietnam), no broadly implemented, internationally accessible vaccine is currently available. Consequently, control continues to depend on early detection, rigorous biosecurity measures, and the culling of infected and exposed animals (1–3). The potential for indirect transmission through contaminated fomites and products underscores the need for strict hygiene and movement control (4). The disease results in significant economic losses due to high mortality rates, stringent trade restrictions, and the substantial costs linked to containment and eradication efforts (5).

Since its first emergence in Asia, ASF has posed considerable challenges to animal health management, threatening food security, destroying livelihoods, and severely impacting pig production systems across the region (3). At least 20 countries and territories in Asia and the Pacific have reported ASF since 2018 (3). ASF was first detected in Cambodia in April 2019 (in Ratanakiri province) and subsequently spread to Tboung Khmum, Svay Rieng, Takeo, and Kandal provinces. These initial outbreaks resulted in the mortality and culling of approximately 3,500 pigs and caused major disruptions to domestic pig production (6, 7). The immediate impact was severe, with nationwide pig production decreasing by approximately 20% in 2019. Following these outbreaks, abattoir-based surveillance conducted across four slaughter facilities in Phnom Penh between October 2019 and January 2020 revealed an ASF seroprevalence of 2.6% (95% CI: 1.6–4.1, *n* = 664) among slaughtered pigs, suggesting limited but persistent virus circulation in the commercial pig population during this period (8).

To prevent further outbreaks on farms, farmers' knowledge, attitudes, and practices (KAP) are recognized as crucial factors influencing their compliance with strict biosecurity measures (3). In practice, better awareness and adherence to good hygiene practices, quarantine protocols, and reporting procedures can substantially reduce virus transmission, whereas misconceptions and poor attitudes may undermine disease control efforts (9). Previous studies conducted in other ASF-affected countries have demonstrated that conventional husbandry practices, economic constraints, and knowledge gaps all contributed to ongoing outbreaks and hindered recovery (10, 11). For instance, a study in Vietnam showed that pig farmers' insufficient knowledge of ASF before the first wave of outbreaks hindered their capacity to react effectively. However, following the outbreaks, improved attitudes toward disease prevention and a stronger willingness to implement biosecurity measures were associated with greater awareness. Moreover, farmers with previous experience managing livestock diseases were more likely to adopt control measures, demonstrating the importance of education and practical exposure in shaping behavior (12).

In Cambodia, pig farming is essential to food security and rural livelihoods. The General Directorate of Animal Health and Production (GDAHP) reported that the country's pig population was approximately 3.4 million in 2022, of which 2.21 million were raised on commercial farms (13). However, Cambodia still relies on official imports of live animals and animal products from neighboring countries, such as Vietnam and Thailand, to meet domestic demand (14). National authorities and development partners have made considerable efforts to disseminate ASF-related information through training sessions, awareness campaigns, and extension services (3). However, it remains unclear how extensively this information has been utilized and understood by pig farmers in Cambodia. Most existing Cambodian studies have focused on pig smallholders, with limited evidence on commercial or contracted farming system under private veterinary supervision, where incentives and compliance mechanisms differ substantially (15). To the best of the authors' knowledge, no published studies have specifically assessed KAP related to ASF among commercial pig producers in Cambodia.

Therefore, this study aimed to assess KAP regarding ASF among commercial pig producers in Kampong Speu and Takeo provinces and to identify factors associated with the implementation of biosecurity practices. Recognizing that knowledge alone does not necessarily translates into behavior change, the KAP findings were interpreted within the framework of behavior-change models that emphasize the roles of capability, opportunity, and motivation in influencing practices (16). The findings are expected to support evidence-based policy formulation and guide the design of context-specific training and risk communication strategies to strengthen ASF preparedness and response within Cambodia's commercial pig sector.

## 2 Materials and methods

### 2.1 Study design and setting

**Figure 1:**
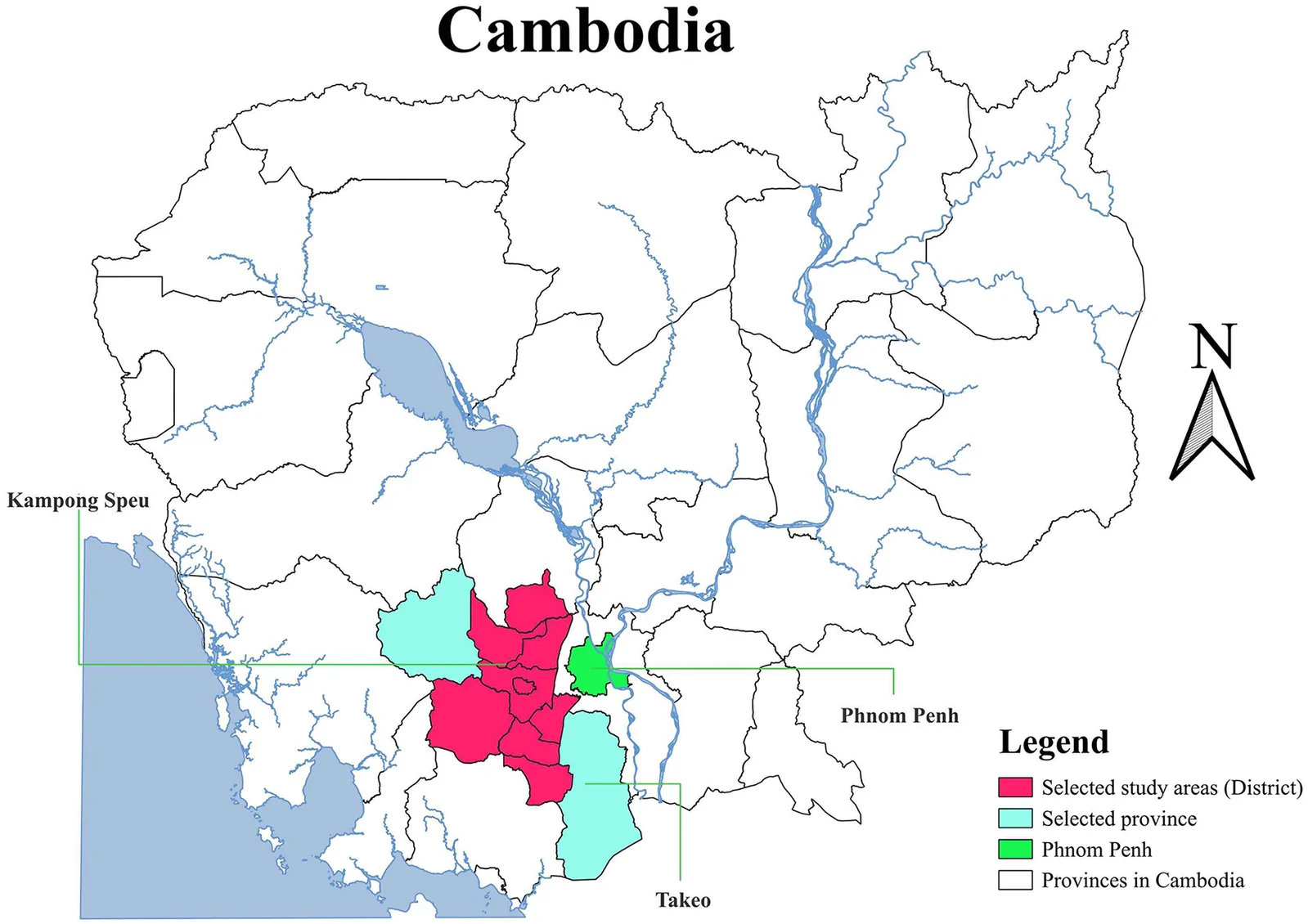
Geographical map of Kampong Speu and Takeo province, Cambodia. The two highlighted provinces represent the study areas where commercial pig farm surveys were conducted from August to October 2024. Provincial boundaries are based on administrative data from the Cambodian Ministry of Interior (2024). Map created using QGIS 3.34 (QGIS Development Team, 2024).

A cross-sectional survey was conducted among commercial pig farms in Kampong Speu and Takeo provinces, Cambodia (Figure 1). These provinces were purposively selected as they represent the highest density of commercial pig farms according to the national report by the GDAHP (13). The three types of commercial farms were categorized as follows: (i) small commercial farms with 100 to less than 1,000 fattening pigs or 50 to less than 200 breeding pigs, (ii) medium-sized farms with 1,000 to less than 5,000 fattening pigs or 200 to less than 500 breeders, and (iii) large farms with more than 5,000 fattening pigs or more than 500 breeders. The study was carried out between August and October 2024 in Kampong Speu and Takeo provinces, Cambodia, two regions with a high concentration of commercial swine production. Study districts were purposively selected through consultation with the Provincial Office of Animal Health and Production (POAHP) and participating swine production companies to ensure representation of the major commercial farm categories operating in the study area. Based on these consultations, eight of the nine districts in Kampong Speu province and one of the ten districts in Takeo province were included, reflecting the geographical distribution and diversity of commercial swine farms. Within the selected districts, participating companies provided lists of eligible farms based on farm availability and farmers' willingness to participate in the study. Company representatives facilitated initial contact with farm owners and accompanied survey teams during field visits to assist with farm access and introductions. To minimize potential response bias, company representatives were not present during interviews and were requested to remain in a separate location out of sight of respondents throughout the data collection process. A survey was conducted by veterinary students and faculty members from the Faculty of Veterinary Medicine at Royal University of Agriculture (RUA). The number of farms surveyed in each district was determined according to the distribution and availability of commercial swine farms within the study area.

### 2.2 Sample size and study design

The sample size was calculated using the formula for estimating a single population proportion (17) assuming a 95% confidence level, 10% absolute precision, and an expected proportion of 0.50. Based on these parameters, a minimum sample size of 96 commercial pig farms was required. In practice, 76 farms were surveyed (67 in Kampong Speu; 9 in Takeo). This reduction was primarily due to logistical constraints, including farmer unavailability during peak production periods, and reluctance to participate among some contracted farms.

### 2.3 Questionnaire development and data collection

A structured questionnaire was designed by researchers from the RUA and subsequently reviewed by researchers from the French Agricultural Research Center for International Development (CIRAD) for clarity. The questionnaire was initially developed in English and subsequently translated into the Khmer. The structure of the questionnaire comprised four main sections totaling 88 questions: (1) socio-demographic and farm characteristics (26 questions); (2) knowledge on ASF disease characteristics and ASF-related training (18 questions); (3) attitudes toward ASF impacts on production, the effectiveness of biosecurity, disease reporting, and perceived ASF risk (16 questions); and (4) practices related to the implementation of biosecurity measures for ASF prevention and control, as well as challenges in ASF management and biosecurity implementation (28 questions). The questionnaire was pilot-tested with two commercial pig producers in Kampong Speu province to evaluate comprehension and timing, resulting in minor revisions. The survey was administered electronically using KoboToolbox (Kobo Inc., 2024) with built-in skip logic and range checks, as well as in a hard copy format depending on the context. Prior to the field survey, four enumerators from the Faculty of Veterinary Medicine, RUA, were trained for 1 day on study procedures, informed consent, and standardized interview administration.

The surveys were conducted under the guidance of the POAHP in each target province, in collaboration with contracted swine companies. Interviews were conducted at various locations, including the POAHP offices, company offices, and farms. For each farm, respondents were selected based on their knowledge of farm biosecurity and ASF, such as the owner, manager, or a designated staff member. Face-to-face interviews were lasted approximately 45–60 min.

### 2.4 Statistical analysis

Data relating to current KAP concerning ASF, the implementation of biosecurity measures for ASF, and key challenges in ASF prevention and control were analyzed using descriptive statistics. Correlations among the KAP categories were assessed using the Chi-square test. The prevalence of high KAP levels was reported with 95% confidence intervals (CI) calculated using the Wilson score interval method.

KAP key individual's answers were scored and categorized following Bloom's cut-off score as indicated in Table 1.

**Table 1:** Awareness evaluation by Bloom cut off point.

Awareness criteria	< 60%	60%−80%	>80%	Remarks
Knowledge	Low	Moderate	High	One mark was awarded for each correct answer, while zero marks were given for incorrect or “do not know”; or “not sure”; or “do not answer” responses
Attitudes	Not concerned	Neutral	Concerned	The individual's attitude response assessment assigned scores ranging from five marks (for “Strongly Agree”) to one mark (for “Strongly Disagree”) for positive statements. For negative statements, reverse scoring was applied
Practices	Poor	Fair	Good	In the individual's practices assessment, positive statement responses were rated from five to one mark, ranging from “Always” to “Never.” Reverse scoring was applied to negative statements

### 2.5 Ethical approval

The study received institutional clearance from the GDAHP, Ministry of Agriculture, Forestry, and Fisheries, Cambodia. Written informed consent was obtained from all participants, and they were informed of their right to withdraw from the study at any time. To ensure confidentiality and protect participant privacy, no personal identifiers were retained in the final dataset.

## 3 Results

### 3.1 General characteristics of farm and producers

A total of 76 participants from contract farms were recruited for this study. The majority of participants were male (79.0%). Most producers had a high level of education, with 64.5% having completed high school or higher education. Most study participants were farm owners (88.2%), and 43.4% reported having 6–10 years of farming experience. Regarding farm characteristics, most operations were medium-scale (61.8%) or large-scale (34.2%), and the majority employed 10 or fewer workers (76.3%). Only a small proportion of farms had on-site veterinarians (10.5%). Fattening pigs was the predominant production activity, accounting for 75.0% of farms. Nearly all producers relied on company technicians for animal health advice (97.4%), whereas provincial veterinarians and on-site veterinarians were consulted much less frequently. Digestive and respiratory disorders were the most commonly reported health issues (Table 2).

**Table 2:** Participant's demographics and farm's characteristics (*N* = 76).

Description	Total participants	
	*n*	%
**Gender**		
Male	60	79.0
Female	16	21.0
**Education**		
Primary school	6	7.9
Secondary school	21	27.6
High school	35	46.1
University/college	14	18.4
**Position in the farm**		
Farm owner	67	88.2
Farm manager	7	9.2
On-site veterinarian	2	2.6
**Experience in current role (years)**		
1–5	17	22.4
6–10	33	43.4
11–15	17	22.4
16–20	7	9.2
>20	2	2.6
**Type of production**		
Fattening	57	75.0
Breeders	15	19.7
Mixed	4	5.3
**Type of farm**		
Contract farm	76	100.0
Farm scale^*^		
Small-scale	3	4.0
Medium-scale	47	61.8
Large-scale	26	34.2
**Workforce (number of employees)**		
≤ 10	58	76.3
11–20	12	15.8
>20	6	7.9
**On-site veterinarian permanently working at farm**		
Yes	8	10.5
**Primary source of advice for animal health**		
Technician (contracted company)	74	97.4
Own experience	22	29.0
Other farms	13	17.1
On-site veterinarian	8	10.5
Provincial/district veterinarian	5	6.6
Swine cooperative	3	4.0
Other	1	1.3
Reported health issues (multiple responses)^**^		
None	2	2.6
Digestive	29	38.2
Respiratory	23	30.3
Reproductive disorder	5	6.6
Skin	2	2.6
Others (convulsion, ASF, FMD, PRRS, etc.)	15	19.7

^*^Farm scale cut-offs as per methods

^**^Multiple-response items use N = 76 as denominator; totals can exceed 100%.

### 3.2 Information, technical advice and training received for ASF of respondents

Table 3 shows that ASF information dissemination, provision of technical advice, and training were predominantly driven by contracted companies, with limited involvement from public veterinary services, universities, and NGOs. Company technicians were the main source of ASF information, representing over half of the reported information channels (56.6%). Provincial and district veterinarians played a very limited role in information dissemination (1.3%). Peer-to-peer information exchange among farmers also contributed to ASF awareness (11.8%). In terms of technical support, the vast majority of respondents (94.8%) relied on company technicians for advice, while only a small proportion received guidance from on-site veterinarians or swine cooperatives (2.6% each). Although most respondents reported having received training on ASF (71.1%), a substantial proportion (28.9%) had not participated in any formal training. Among trained respondents, contracted companies were the principal training providers, accounting for 90.7% of all training activities. However, the majority of producers had received training for more than 2 years prior to the survey. Farmers recalled receiving information on ASF symptoms, disease prevention measures, and biosecurity practices during these company-organized events.

**Table 3:** Sources of information, technical advice, and training related to ASF among respondents (*N* = 76).

Description	Total participants	
	*n*	%
**Information channels**		
Company technicians	43	56.6
Social media	13	17.1
Other farmers	9	11.8
Mass media (TV, radio, newspaper)	5	6.6
Provincial/district veterinarians	1	1.3
Others	5	6.6
**Technical advice providers**		
Company technicians	72	94.8
On-site veterinarians	2	2.6
Swine cooperative	2	2.6
**Received training on ASF**		
Yes	54	71.1
**Time received the training (months)**		
6–12	4	7.4
>12–24	12	2.2
< 24	38	70.4
**Training provider institutions**		
Contracted companies	49	90.7
Government	10	18.5
University	1	1.9
NGOs	1	1.9

Multiple responses allowed unless stated.

### 3.3 Knowledge of swine producers on ASF

Knowledge scores were expressed as mean values and classified using Bloom's cut-off criteria into poor (< 60%), moderate (60%−80%), and good (>80%) levels. Table 4 presents respondents' knowledge of ASF across disease characteristics, clinical signs, transmission routes, diagnostic approaches, and control and prevention measures. Overall, respondents demonstrated a generally good understanding of the basic characteristics of ASF, with high proportions recognizing the disease as incurable (92.1%), preventable (96.1%), and notifiable (93.4%); however, important knowledge gaps were evident, as only 54.0% correctly identified ASF as a viral disease and only 31.6% acknowledged the absence of an effective vaccine. Knowledge of clinical signs was uneven, with red skin discoloration being the most frequently reported symptom (89.5%), whereas recognition of other key signs such as high fever (68.4%) and bloody diarrhea (19.7%) was substantially lower. In relation to transmission, most producers were aware of common pathways, including direct contact between pigs (84.2%), fomites and vehicles (82.9%), and contaminated pork products or carcasses (77.6%); in contrast, fewer respondents identified swill feeding (46.1%) and contact with wild swine (13.2%) as risk factors. Regarding diagnosis, the majority relied on observation of clinical signs (86.8%) and laboratory confirmation (75.0%), while knowledge of post-mortem examination (7.9%) and epidemiological history or tracing (2.6%) was minimal. Concerning control and prevention, biosecurity and hygiene measures were widely recognized (96.1%), but awareness of additional control strategies, including swine movement restrictions (38.2%) and emergency preparedness (4%), remained limited.

**Table 4:** Knowledge items on African swine fever (*N* = 76).

Description	Knowledge score		
	*n*	%	CI 95%
**General knowledge on ASF characteristics**			
ASF is an uncurable disease	70	92.1	83.8–96.3
ASF is a preventable disease	73	96.1	89.0–98.6
ASF is a notifiable disease	71	93.4	85.5–97.2
ASF virus is very resistant and survives long periods in various conditions	63	82.9	72.9–89.7
ASFV can remain viable for long period in nasal fluids, urine and feces	65	85.5	75.9–91.7
ASF is a disease cause by a virus	41	54.0	42.8–64.7
No effective vaccine to prevent ASF	24	31.6	22.2–42.7
**Knowledge on ASF symptom in swine**			
Presence of red loose skin coloration in the ventral abdomen, tips of ears or tail or distal limb	68	89.5	85.6–94.6
High fever	52	68.4	57.3–77.8
Difficult to breath	16	21.1	13.4–31.5
Abortion or sudden death after birth	16	21.1	13.4–31.5
Diarrhea (sometimes mix with blood)	15	19.7	12.3–30.0
Vomiting	14	18.4	11.3–28.6
**Knowledge of ASF transmission**			
Direct contact with infected swine	64	84.2	74.4–90.7
Vehicles or equipment spreading the germs	63	82.9	72.9–89.7
Contact with contaminated pork products/carcasses	59	77.6	67.1–85.5
Visitors spreading the germs (e.g., swine traders)	55	72.4	61.4–81.2
Biting insects/rodents (ticks, flees…)	54	71.1	60.0–80.0
Feeding of infected/contaminated swine meat/swill/offal to swine	35	46.1	35.3–57.2
Direct contact with infected wild swine	10	13.2	7.3–22.5
**Knowledge of ASF diagnosis**			
Base on the clinical signs	66	86.8	77.4–92.7
Base on the lab test (PCR, ELISA, Histopathology)	57	75.0	64.2–83.3
Post-mortem (Necropsy)	6	7.9	3.7–16.2
History investigation (Herd, swine or pork product movement...)	2	2.6	0.7–9.1
**Knowledge on control and prevention measures for ASF**			
Implement strict good hygiene	73	96.1	89.0–98.6
Restrict visitors and limit workers from moving between pens	49	64.5	53.3–74.3
Restrict the movement of swine and swine products	29	38.2	28.1–49.4
Avoid feeding swine with uncooked food waste or swill	13	17.1	10.3–27.1
Education and training: transmission and prevention	8	10.5	5.4–19.4
Emergency preparedness: response plan for ASF outbreaks	3	4.0	1.4–11.0
Regularly monitor swine for signs of ASF and report any suspected cases	3	4.0	1.4–11.0

### 3.4 Attitude of swine producers toward ASF

Attitude scores were expressed as mean values and classified using Bloom's cut-off criteria into poor (< 60%), moderate (60%−80%), and good (>80%) levels. As shown in Table 5, swine producers had a strong attitude toward ASF control, with a mean score of 89.4% (ranging from 80.6% to 94.6%) and with very high endorsement of biosecurity effectiveness and movement/visitor control (most items >90%). However, reporting of suspected outbreaks (72.4%) and accurate public-health understanding (68.4% believed that ASFV does not affect human health) showed lower endorsement, which could have an impact on timely notification and risk communication. Most swine producers considered their operation to be low risk (65.8%), only 11.8% still classified their farms as high risk, recognizing ASF as a highly contagious and deadly disease (data not shown). This might highlight a potential optimism bias.

**Table 5:** Attitudes toward African swine fever risk and control (*N* = 76).

Statement^*^	Attitude score^**^,^***^		
	*n*	%	CI 95%
Biosecurity is very effective for preventing ASF	74	97.4	90.9–99.3
Admission of visitors into pens should be prohibited	74	97.4	90.9–99.3
Movement of people in and out of the farm areas increases the risk of transferring ASFV into holding units	73	96.1	89.0–98.6
Quarantine of new swine could prevent ASF in the farm	72	94.7	87.2–97.9
Movement of other animals in and out of the farm areas increases the risk of transferring ASFV into holding units	72	94.7	87.2–97.9
ASF is an important disease that affects swine production	71	93.4	85.5–97.2
Swine feed delivery to the farm could cause ASF	71	93.4	85.5–97.2
Workers and visitors must be aware of biosecurity measures in place to minimize the risk of ASFV introduction into the farm	71	93.4	85.5–97.2
Movement of vehicles in and out of the farm areas increases the risk of transferring ASFV into holding units	70	92.1	83.8–96.3
Pest control is recommended to minimize the risk of ASF	68	89.5	80.6–94.6
Build pen with double fence to avoid contact with other swine is recommended	66	86.8	77.4–92.7
There is no effective vaccine to prevent ASF	62	81.6	71.4–88.7
You should report ASF outbreaks on your farm to local authorities, or veterinarians	55	72.4	61.4–81.2
Infected swine with ASFV is not harmful to public health	52	68.4	57.3–77.8
**Mean** ^*^	**68**	**89.4**	**80.6–94.6**

^*^Scoring: Positively worded items scored 5 = Strongly agree; 1 = Strongly disagree; reverse-coded where needed; summed and rescaled to 0–100.

^**^Bloom cut-offs (Attitudes): < 60% = Not concerned; 60– < 80% = Neutral; ≥80% = Concerned.

^***^Favorable proportion: “Agree/Strongly agree” collapsed for display. ^****^95% CIs for proportions use Wilson method; denominator N = 76 unless noted. ^*^Rate of mean in agreement.

### 3.5 Practice of swine producers regarding ASF

Table 6 displays the frequency of biosecurity practices used at swine farms, with 76 participants responding. Biosecurity practices on swine farms showed strong compliance, with 100% adherence to vehicle disinfection, 98.7% for equipment disinfection and dead animal disposal, and 97.4% for hygiene measures for workers and service providers. Moderate compliance was observed in rodent control (86.8%) and insect control (82.9%), as well as quarantining new swine (76.3%) and implementing biosecurity measures for visitors and traders, respectively (86.8% and 82.9%). However, lower compliance rates were observed in wild bird control (69.7%), downtime between batches (68.4%), and reporting ASF cases (56.6%), indicating areas that require improvement.

**Table 6:** Frequency of applying biosecurity measures (adherence).

Description biosecurity measures	Practice score		
	*n*	%	CI 95%
Disinfect vehicles (staff, feed truck, etc.) before entering the swine farm	76	100.0	95.2–100
Not sharing equipment/tools between farm or house/pen without disinfection	75	98.7	92.9–99.8
Depose dead animals (buried, burned, etc.) according to the proper guideline	75	98.7	92.9–99.8
Apply cleaning and disinfection after raising each batch	75	98.7	92.9–99.8
Allow workers to enter the swine farm by taking biosecurity measures including showering disinfects or changing clothes and wearing boots	75	98.7	92.9–99.8
Allow service providers (e.g., feed distributors, medicine distributors) to enter the swine farm by taking biosecurity measures including showering disinfect or changing clothes and wearing boots	74	97.4	90.9–99.3
Not allowed to bring the pork and other swine products to the farm	74	97.4	90.9–99.3
Do not allow other animals to the swine's house/pen	74	97.4	90.9–99.3
Allow veterinarians to enter the swine farm by taking biosecurity measures including showering disinfects or changing clothes and wearing boots	73	96.1	89.0–98.6
Dedicate a specific loading area to deliver swine	71	93.4	85.5–97.2
Forbidden workers to raise swine at home	69	90.8	82.2–95.5
Apply rodent control on your farm	66	86.8	77.4–92.7
Allow traders to enter the swine farm by taking biosecurity measures including showering/disinfect or changing clothes and wearing boots	66	86.8	77.4–92.7
Allow visitors to enter the swine farm by taking biosecurity measures including showering/disinfecting or changing clothes and wearing boots	63	82.9	72.9–89.7
Apply insect control on your farm	63	82.9	72.9–89.7
Quarantine the new swine before moving the existing swine's house/pen	58	76.3	65.6–84.5
Apply wild birds' control on your farm	53	69.7	58.7–78.9
Maintain a minimum downtime period of 2 weeks between batches	52	68.4	57.3–77.8
Report of ASF suspicious cases on your farm to local authorities, or veterinarians	43	56.6	45.4–67.1
**Mean** ^*^	**67**	**88.3**	**79.0–93.6**

N = 76 unless stated. ^*^Rate of mean in routines.

### 3.6 Correlation of knowledge with attitudes and practices

There was no statistically significant association between knowledge level and attitude (*p* > 0.05). The very high proportion of respondents categorized as having a “Concerned” attitude across all knowledge levels, particularly among those with poor knowledge (67.1%) together with the small proportion of respondents with good knowledge, substantially limited the ability to detect any potential association (Table 7).

**Table 7:** The correlation between knowledge and attitudes (percentage, *N* = 76).

Knowledge/Attitude	Not concerned % (CI 95%)	Neutral % (CI 95%)	Concerned % (CI 95%)
Low	1.3 (0.23–7.08)	5.3 (2.07–12.8)	67.1 (55.9–76.6)
Moderate	1.3 (0.23–7.08)	3.9 (1.35–11.0)	19.7 (12.3–30.0)
High	0.0	0.0	1.3 (0.23–7.08)
**Chi_square value**	**2.18**		
***p*** **_value**	**0.70**		

Knowledge level also did not appear to have a strong influence on practice, as no significant relationship was observed between knowledge and practice (*p* = 0.71). Notably, approximately 40% of respondents with poor knowledge were already demonstrating good practices. Similarly, good practices were commonly observed even among respondents with limited knowledge (Table 8).

**Table 8:** The correlation between knowledge and practices (percentage, *N* = 76).

Knowledge/Practice	Poor % (CI 95%)	Fair % (CI 95%)	Good % (CI 95%)
Low	11.8 (6.36–21.0)	22.4 (14.5–32.9)	39.5 (29.2–50.7)
Moderate	6.6 (2.84–14.5)	7.9 (3.67–16.2)	10.5 (5.43–19.4)
High	0.0	1.3 (0.23–7.08)	0.0
**Chi_square value**	**2.14**		
***p*** **_value**	**0.71**		

Although the distribution of percentages suggests that higher levels of concern may be associated with better practices, the Chi-square test showed that this pattern was not statistically significant (*p* > 0.05). Therefore, the null hypothesis could not be rejected, indicating insufficient statistical evidence to support a true association between attitude and practice in the study population (Table 9).

**Table 9:** The correlation between attitude and practice (percentage, *N* = 76).

Attitude/Practice	Poor % (CI 95%)	Fair % (CI 95%)	Good % (CI 95%)
Not concerned	0.0	2.6 (0.72–9.10)	0.00
Neutral	1.3 (0.23–7.08)	3.9 (1.35–11.0)	3.9 (1.35–11.0)
Concerned	17.1 (10.3–27.1)	23.7 (15.3–34.4)	47.4 (36.5–58.4)
**Chi_square value**	**5.5**		
***p*** **_value**	**0.24**		

Tables 10–13 emphasized the respondent's gender, education, experience, and production system influence on KAP. The data showed minor variations in KAP, and overall scores between male and female respondents. However, these differences were not statistically significant. Since all *p-values* exceed 0.05, either gender, education, experience, and production system did not appear to a significant role in influencing knowledge, attitude, or practices in this study.

**Table 10:** Respondent gender influenced by knowledge, attitudes, and practices.

Description	Female (%)	Male (%)	*p-*Value
Knowledge	46.24	52.69	**0.085**
Attitude	88.84	89.52	**0.870**
Practice	77.11	73.50	**0.330**
**Mean score** ^*^	**70.73**	**71.91**	**0.556**

^*^Comparison of mean KAP scores by respondent gender.

**Table 11:** Respondent education influenced by knowledge, attitudes, and practices.

Description	Primary school	Secondary school	High school	University	*P*-value
Knowledge	50.91	50.79	50.69	53.93	0.889
Attitude	90.48	89.46	88.78	90.31	0.986
Practice	64.53	76.51	74.23	75.12	0.262
**Mean score** ^*^	**68.64**	**72.25**	**71.23**	**73.12**	**0.605**

^*^Comparison of mean KAP scores by respondent education.

**Table 12:** Respondent experience influenced by knowledge, attitudes, and practices.

Description	1–5 (years)	6–10 (years)	11–15 (years)	16–20 (years)	>20 (years)	*P*-value
Knowledge	47.42	53.61	51.47	50.07	50.12	0.654
Attitude	92.02	87.01	89.92	91.84	92.86	0.793
Practice	78.3	72.31	73.51	76.8	69.58	0.579
**Mean score** ^*^	**72.58**	**70.98**	**71.63**	**72.9**	**70.85**	**0.942**

^*^Comparison of mean KAP scores by respondent education.

**Table 13:** Respondent production system influenced by knowledge, attitudes, and practices.

Description	Small-scale (%)	Medium-scale (%)	Large-scale (%)	*P*-value
Knowledge	50.48	50.5	53.03	0.745
Attitude	92.86	90.03	88.71	0.752
Practice	72.94	74.45	82.2	0.510
**Mean score** ^*^	**71.23**	**71.66**	**75.18**	**0.676**

^*^Comparison of mean KAP scores by respondent education.

## 4 Discussion

### 4.1 Producers' knowledge of ASF

This study was conducted to assess the KAP related to ASF among commercial pig producers in Kampong Speu and Takeo provinces, Cambodia, and to identify factors influencing the implementation of biosecurity measures. Understanding KAP is essential for strengthening ASF prevention and control, as farmers' perceptions and management behaviors directly influence disease transmission risk and the effectiveness of biosecurity interventions. Recognizing that knowledge alone may not necessarily lead to behavioral change, the study interpreted the KAP findings within the framework of behavior-change models that emphasize the roles of capability, opportunity, and motivation (16). The study revealed that biosecurity implementation and ASF-related support were largely driven by contracted pig production companies, which played a dominant role in information dissemination, technical guidance, and training activities. These findings highlight the importance of institutional support systems in shaping disease prevention practices among commercial pig producers. The results provide important evidence for policy development and may support the design of context-specific training, communication, and biosecurity strategies aimed at improving ASF preparedness and response in Cambodia's commercial pig sector.

Overall, pig producers demonstrated a moderate level of awareness of ASF characteristics, with a mean knowledge score of 76.5%, and individual scores ranging from 31.6% to 96.1% (Table 4). High awareness of ASF's incurability (92.1%), preventability (96.1%), notifiability (93.4%), and virus persistence and survivability (84.2%) indicate successful dissemination of basic ASF information. Similar findings have been reported in Vietnam, Uganda and Ukraine, where producers also demontrated strong fundamental awareness of ASF (10, 12, 18). However, only 54.0% of respondents correctly identified ASF as a viral disease, and only 31.6% were aware that no commercially available vaccine existed at the time of the study. These findings suggest important knowledge gaps regarding ASF etiology and prevention. While respondents may still recognize ASF as a serious disease threat, limited understanding of its cause and the absence of a vaccine may affect how they perceive the importance of strict biosecurity and timely disease reporting, which remain the primary strategies for preventing ASF introduction and spread (19). Therefore, these results highlight the need for continued education and veterinary engagement to strengthen ASF-related knowledge and support the translation of knowledge into appropriate preventive behaviors.

Although the majority of producers received ASF training (71.1%), knowledge of ASF symptoms was also variable. While visible signs such as red skin coloration were well-recognized (89.5%) and high fever (68.4%) were well-recognized, less obvious but important clinical signs, such as vomiting and bloody diarrhea, were correctly identified by fewer than 20% of respondents (Table 4). Knowledge on ASF symptoms among Cambodian producers were comparable to that reported among Vietnamese and Nigerian farmers (12, 20). However, these knowledge gap could delay disease detection and reporting, undermining containment efforts (21). A study in England reported that swine farmers with limited knowledge of ASF clinical signs and who perceived ASF as less concerning than other swine diseases, were less likely to consider the possibility of an ASF outbreak occurring on their farms. Furthermore, those who are unaware of ASF outbreaks in other countries, perceived negative consequences from false-positive reporting, and found reporting procedures complexes were less inclined to report suspected ASF cases (22).

Regarding transmission routes, most producers were well-aware of direct contact with infected swine (84.2%) and vehicle contamination (82.9%); while demonstrating moderately aware of transmission through contaminated pork products, visitors, and insects or rodents (71.1%−77.6%). However, lower awareness of indirect routes such as feeding contaminated meat (46.1%) and contact with wild swine (13.2%) was of concern. Similar knowledge gaps have been reported among swine producers in Nigeria (20), suggesting that awareness of less obvious transmission routes remains a challenge across different production settings. Diagnostic knowledge was similarly mixed. While clinical signs and laboratory testing were widely acknowledged as diagnostic approaches (86.8 and 75%, respectively), few producers recognized the diagnostic value of post-mortem examination or herd history. This finding highlights the need for producer education on comprehensive disease investigation approaches.

Preventive measures were generally well-understood, with nearly all producers recognizing the important of biosecurity and hygiene practices (96%). However, only 38% were aware of movement restrictions, and even fewer respondents (4%) recognized the important of emergency preparedness procedures. A study conducted in Nigeria found that 68.8% of pig farmers implemented strict movement restrictions on their farms, and 48.6% consistently quarantined newly acquired pigs before introducing them into their herds. Despite these measures, most pig farms were still inadequately prepared to prevent the introduction of ASF (20). Movement restrictions and emergency preparedness are essential components of both national and farm-level ASF response plans and should therefore be emphasized in extension and risk communication programs (23).

### 4.2 Producers' attitudes toward ASF

A significant proportion of producers (65.8%) perceived their farms as being at low risk for ASF introduction, despite evidence of inconsistent biosecurity practices. This misalignment suggests a degree of complacency that could undermine preventive efforts. Although only 11.8% viewed their farms as high risk, the overall perception of ASF severity remained high (89.4%), indicating substantial concern regarding the potential impacts of the disease. Similar finding has been reported in Uganda (24). However, many producers were still not willing to report the ASF outbreaks on their farms to local authorities or veterinarians (27.6%). Considering that pig production was the sole source of income for many respondents, the potential economic consequences of reporting may have discouraged outbreak notification.

Finally, some producers held the misconception that ASFV poses a public health threat. Although ASF does not infect humans, such misunderstandings could lead to unnecessary fear or stigma and should be addressed in awareness campaigns. This finding is consistent with studies conducted in Uganda and Nigeria (20, 24). Misunderstandings regarding ASF's impact on human health remain a barrier to effective disease management. As these misconceptions may reduce trust in authorities and disease control messages, it is essential to reinforce the fact that ASF is non-zoonotic disease that affects swine exclusively (10).

### 4.3 Producers' practice regarding ASF

Compliance with specific biosecurity measures revealed a tiered pattern. High compliance was observed for disinfection protocols and visitor clothing requirements (over 85%), aligning with recommended best practices (25). Moderate implementation was found for practices such as feed equipment control and staff movement restrictions (60%−72%). However, critical areas such as animal quarantine, manure management, and visitor tracking showed lower compliance, indicating potential entry points for ASFV. Particularly low adoption of visitor shower facilities and biosecurity signage may reduce the effectiveness of otherwise strong hygiene measures.

Despite generally moderate knowledge scores, statistical analyses (Tables 7–9) showed no significant relationship between knowledge and either attitude or biosecurity practices (*p* > 0.05). This finding is consistent with previous studies suggesting that knowledge alone does not necessarily translate into behavior change (25). Similarly, studies in Uganda and Vietnam reported strong ASF awareness among producers but inconsistent implementation of biosecurity practices (10, 12). In contrast, a study in Japan found that higher levels of knowledge and more positive attitude were associated with improved biosecurity practices on swine farms (16). These mixed findings suggest that behavioral changes may be associated with not only awareness but also structural support, incentives, and culturally tailored communication strategies. For example, Swedish swine farmers faced financial and practical barriers to implement recommended biosecurity measures (26). Furthermore, the cost of such measures has been shown to influence the farmer's motivation in both Japan (16) and Uganda (27).

Although compliance with several biosecurity measures was high (>85%), significant knowledge gaps were still identified among respondents, particularly regarding the recognition of ASF as a viral disease (54%), the absence of an effective vaccine for ASF prevention (32%), and emergency preparedness measures (4%). These findings suggest that producers operating under contract farming arrangements with pig production companies are required to comply with standardized biosecurity measures and management protocols mandated by the contracting industries, regardless of their individual level of ASF-related knowledge. Consequently, adherence to biosecurity practices among contract farms may be driven more by institutional requirements and company supervision than by farmers' personal awareness or understanding of disease prevention. Furthermore, contract farming systems differ structurally from traditional smallholder production systems commonly described in previous studies. Contract farms generally operate under stricter production guidelines, receive continuous technical support from company technicians, and are subject to mandatory biosecurity compliance and monitoring. In contrast, smallholder farms often rely on individual decision-making, have more variable access to veterinary services and training, and may implement biosecurity measures inconsistently. Therefore, the comparatively high level of biosecurity implementation observed in this study may reflect the organizational and regulatory characteristics of contract farming systems rather than solely the knowledge or attitudes of producers. Previous studies have reported an association between a deeper understanding of ASF, more positive attitudes toward its prevention and control, and a greater likelihood of adopting ASF biosecurity practices (12). Previous studies have highlighted a positive association between ASF knowledge and favorable attitudes toward biosecurity measures. Additionally, previous studies have reported that farmers with prior experience of infectious swine disease outbreaks were more likely to report implementing ASF biosecurity protocols. These findings support the potential value of strengthening community education campaigns and increasing financial assistance and support for swine farmers to encourage the implementation of effective biosecurity measures (12).

Sociodemographic variables such as gender, education, experience, and production system did not significantly influence KAP outcomes (Tables 10–13). This suggests a relatively uniform distribution of knowledge and practices across these groups, likely due to shared exposure to similar information sources or homogeneity in farming contexts. It also implies that interventions may not need to be heavily stratified by these demographics but should instead focus on strengthening farm-level biosecurity behaviors regardless of producer background or contractual requirements imposed by pig production companies.

At present, the implementation of proper biosecurity measures and early detection remains the most effective strategy for preventing and controlling ASF outbreaks on swine farms (23, 28). Moreover, producers' perceptions of risk and the practicality of implementing biosecurity measures are key factors influencing their adoption, indicating that enhanced outreach and education efforts could promote higher levels of compliance (29). Encouraging practices such as quarantine, biosecurity signposting, and proper manure practices may necessitate regulatory frameworks, veterinary oversight, and subsidized biosecurity tools observed in Vietnam's national plans (30). The study by Klein et al. (31) underscores the vital role of veterinary officials and farm veterinarians as primary sources of biosecurity guidance, highlighting the need for clear and consistent biosecurity standards. It also emphasizes the importance of fostering stronger collaboration between swine farmers and these experts through joint decision-making tailored to the specific circumstances of individual farms. Furthermore, a study in China examined the factors affecting farm biosecurity and found that technical training, farm size, income share, production organization, and government inspections significantly influenced the level of biosecurity practices adopted (32). Similarly, a study in Cameroon indicated that inadequate implementation of biosecurity measures, together with insufficient training, has contributed to the enzootic presence and continued spread of ASFV. Therefore, providing swine farmers in Cameroon with appropriate training on ASFV awareness and its impact on swine production remains essential (33). In line with these findings, our study revealed that 88.3% of producers implemented specific biosecurity measures in accordance with established guidelines, as they operated within contract farming systems linked to the swine production companies. Moreover, 94.8% of producers received technical support and veterinary supervision from contract swine companies. In contrast, a study conducted in Uganda evaluated the impact of a participatory training of swine farmers' related to ASF biosecurity control in two districts of Uganda. The finding indicated that knowledge was not the primary limiting factor effecting the adoption of biosecurity interventions (9). The differences between the Ugandan study and our findings may be explained by the distinct production systems involved. Producers in our study were required to comply with biosecurity protocols established by the contracting companies and were subject to ongoing technical supervision. In contrast, the Ugandan farmers primarily operated independently owned farms and therefore had greater autonomy in deciding whether and how to implement biosecurity measures. Consequently, biosecurity compliance among contract farms may be influenced more by institutional requirements and company oversight than by knowledge alone.

### 4.4 Study limitation

The findings of this study should be interpreted in light of several limitations. First, the convenience sampling approach, while pragmatic given the lack of formal farm registries and the challenges in accessing independent commercial farms, may have introduces potential selection bias. Farms affiliated with contracted companies may differ systematically from independent farms in terms of management practices, biosecurity implementation, and access to veterinary services, as observed in both European and Southeast Asian livestock production systems (34, 35). Second, the unequal inclusion of districts between provinces (eight in Kampong Speu vs. one in Takeo) reflects the uneven geographical distribution of commercial pig farming, with Kampong Speu having a substantially higher farm density. Third, although the study initially aimed to survey 96 farms, only 76 farms were included in the final analysis. This smaller sample size may have reduced the statistical power of the analysis and limited the ability to detect associations between farmer characteristics, ASF-related knowledge and attitudes, and reported biosecurity practices. Fourth, the survey relied on self-reported information from farm operators, which may have introduced reporting bias, including social desirability bias, whereby respondents may have overreported compliance with recommended biosecurity practices. Although measures were implemented to minimize company influence during interviews, these potential sources of bias should be considered when interpreting the results. Additionally, further validation of the questionnaire and reliability testing of the Bloom's cut-off score classification should be considered in future studies. Therefore, the findings should be interpreted as baseline evidence from participating commercial swine farms rather than as fully representative of all commercial farms in the study provinces. Nevertheless, the study provides useful preliminary data on ASF-related knowledge, attitudes, and biosecurity practices in a context where such evidence remains limited.

## 5 Conclusion

This study revealed that while swine producers generally possess a moderate understanding of ASF and demonstrate high awareness of basic disease characteristics, critical knowledge gaps remain, particularly regarding ASF's viral nature, the absence of a vaccine, and less obvious transmission routes. Swine producers demonstrated positive attitudes toward biosecurity measures for ASF. Despite high reported compliance with most biosecurity practices, key areas such as quarantine, manure management, and emergency preparedness were weakly implemented. Importantly, knowledge did not significantly correlate with attitudes or practices, underscoring that awareness alone is insufficient to drive behavioral change. Structural barriers, financial constraints, and risk misperceptions continue to hinder effective ASF control. Strengthening producer education, improving collaboration with veterinary experts, and providing financial and technical support, especially through cooperative and contract farming models, are essential for advancing ASF prevention and control efforts in the country.

## Data Availability

The original contributions presented in the study are included in the article/supplementary material, further inquiries can be directed to the corresponding author.
